# Sobrevivência com CDI na Insuficiência Cardíaca: Realmente necessário ou Apenas um Viés de Seleção?

**DOI:** 10.36660/abc.20250142

**Published:** 2025-03-27

**Authors:** Fernanda Almeida Andrade

**Affiliations:** 1 Hospital das Clínicas da Faculdade de Medicina da Universidade de São Paulo Instituto do Coração São Paulo SP Brasil Instituto do Coração do Hospital das Clínicas da Faculdade de Medicina da Universidade de São Paulo, São Paulo, SP – Brasil; 2 Universidade Federal de São Paulo Escola Paulista de Medicina São Paulo SP Brasil Escola Paulista de Medicina da Universidade Federal de São Paulo (EPM/UNIFESP), São Paulo, SP – Brasil

**Keywords:** Desfibriladores Implantáveis, Insuficiência Cardíaca, Prevenção Primária

Na prática clínica atual, a avaliação de candidatos à terapia primária com cardiodesfibrilador implantável (CDI) exige a consideração de fatores adicionais além da fração de ejeção do ventrículo esquerdo (FEVE), a fim de selecionar com maior precisão os pacientes que realmente se beneficiarão do dispositivo. Evidências recentes sugerem uma redução na incidência de morte súbita cardíaca em indivíduos com FEVE reduzida, associada a uma crescente heterogeneidade dos mecanismos fisiopatológicos envolvidos. Entretanto, diante dos avanços nas abordagens farmacológicas com eficácia na redução da morbimortalidade na insuficiência cardíaca (IC) a decisão sobre a indicação do CDI na prevenção primária tornou-se mais complexa e com necessidade de buscar estratégias inovadoras para a estratificação de risco desses casos.^1^

Tecnicamente, o CDI para prevenção primária é indicado em pacientes com FEVE ≤ 35% e IC classe II-III da *New York Heart Association* (NYHA), apesar de pelo menos três meses de terapia médica otimizada, e então já cabem algumas críticas: a) os estudos que fundamentam essa recomendação têm mais de 20 anos, b) a população com FEVE reduzida representa um grupo heterogêneo, c) diferentes níveis de risco para taquicardia ventricular/fibrilação ventricular (TV/FV) associada ao substrato arritmogênico *versus* mortalidade não arrítmica, ou seja, relacionada à presença de comorbidades e declínio da função cardíaca, e d) implementação de novos tratamentos e melhor manejo da doença.^1 - 3^

O estudo de Başkurt et al.^2^ traz um resultado baseado na análise retrospectiva de 228 pacientes com IC submetidos ao implante de CDI para prevenção primária no período de 62 meses e quando analisamos as taxas de mortalidade entre pacientes com (29,4%) e sem (26%) CDI, houve pior desfecho (óbito) no grupo controle [p < 0,05 (IC 95%)], e embora a longo prazo não houve diferença estatística, a sobrevida foi numericamente melhor no grupo CDI. Esse achado pode ser atribuído ao fato de que os implantes de CDI foram realizados em pacientes com pior condição clínica e também pelo fato de possuírem um acompanhamento regular devido ao controle do dispositivo.^2^ Dados similares foram obtidos através de uma metanálise que avaliou 12 ensaios clínicos randomizados por 20 anos (n 40.195), onde a incidência anual de morte súbita foi de 6,5% no estudo RALES (o primeiro desse período) e de 3,3% no estudo mais recente, PARADIGM-HF. O dado estatístico confere uma redução de 44% na taxa de morte súbita ao longo dos anos [HR 0,56, IC 95% 0,33-0,93, p = 0,03].^3 , 4^ Outros trabalhos também demonstraram achados similares.^4 - 7^

## Preditores de mortalidade: O papel da avaliação clínica pormenorizada

A relevância de uma boa consulta médica deve ser rememorada quando trata-se da avaliação desses casos, através de uma anamnese conseguimos extrair possíveis preditores de mortalidade, como por exemplo: idade, FEVE, valor de BNP e/ou NT-proBNP, doença arterial coronariana (DAC), diabetes mellitus, insuficiência renal crônica, ritmo basal, hospitalização por IC e NYHA >2. A partir do descrito, o trabalho de Başkurt et al.^2^ traz valores como BNP > 508,5 pg/mL (ROC: S 69% e E 69%), FEVE < 24,5% (ROC: S 54% e E 63%) e idade > 68,5 anos (ROC: S 62% e E 62%) e hospitalização por descompensação como fatores independentes de mortalidade por todas as causas; já a DAC não foi um fator de risco independente.^2 , 3^ Estudos similares, como por exemplo, Bilchick et al.^5^ que analisou uma coorte expressiva (n. 45.000) também conferiram dados semelhantes, onde o paciente mais crítico e com mais comorbidades possuem maior risco de desfecho negativo.^5^

Destarte, a necessidade de um escore de risco vem sendo discutido e validados ao decorrer do tempo, por exemplo: *MADIT-II ICD Risk Stratification Score* e o *Seattle Heart Failure Score* mas há diferenças importantes entre os dois: presença de terapia de ressincronização associada (modelo de Seattle não inclui), diferença na definição de arritmia grave e morte arrítmica ( *MADIT-ICD Benefit Score* usa arritmias potencialmente fatais como marcador de morte arrítmica, enquanto outros estudos utilizaram uma distinção entre morte súbita e não súbita) e validação atualizada com coorte contemporânea. O escore de benefício MADIT-ICD proposto em 2021 vem para melhorar a estratificação de risco, visando auxiliar a decisão clínica individualizada através da identificação de candidatos à implantação primária de CDI com maior potencial de benefício para a sobrevida, ou seja, aqueles em que o risco previsto de TV/FV é superior ao risco concorrente de mortalidade não arrítmica.^8^

## Limitações e perspectivas futuras

Contra fatos não há argumentos, as taxas de choques e a mortalidade em pacientes com CDI diminuíram devido ao progresso do sistema de saúde, as terapias farmacológicas para IC e ao acesso mais facilitado ao médico. A indicação do CDI para prevenção primária tem sido questionada, temos "novas" drogas como o inibidor do cotransportador de sódio e glicose tipo 2 e inibidor do receptor de angiotensina-neprilisina que revolucionaram a terapêutica da IC. Atualmente, muitos pacientes com CDI nunca recebem um choque, em contrapartida, tem muitos com choques inapropriados e outras intercorrências sem o dito benefício em sobrevida.^4 , 9 , 10^

As diretrizes poderão necessitar de revisão à medida que novos ensaios multicêntricos e randomizados sejam conduzidos, especialmente aqueles que avaliem populações tratadas com abordagens terapêuticas mais recentes em combinação. Embora a recomendação para a implantação do CDI na prevenção primária da IC não isquêmica tenha sido reduzida nas diretrizes mais recentes, ela permanece como indicação de classe 1 para pacientes com IC de origem isquêmica. Nos estudos que sustentam o benefício do CDI na prevenção primária dessa população, a estratificação dos pacientes foi realizada por meio de testes eletrofisiológicos, e a adesão à terapia médica otimizada (nas diretrizes definida como o uso de betabloqueadores, inibidores da ECA/BRA e antagonistas da aldosterona) era limitada; e ainda hoje as diretrizes internacionais não exigem o uso das "novas" drogas como critério obrigatório. Contudo, a utilização rotineira de testes eletrofisiológicos pré-implante não faz parte da prática clínica habitual. O *MADIT-ICD Benefit Score* surge para auxiliar a tomada de decisão compartilhada entre médico e paciente, por meio de uma avaliação personalizada e integrada do risco concorrente de TV/FV vs. mortalidade não arrítmica em candidatos à terapia com CDI.^1 , 9 , 10^

Concluindo, a utilização de dados provenientes de ensaios clínicos randomizados multicêntricos representa um avanço significativo na compreensão dos benefícios do CDI. No entanto, é necessário reconhecer que os participantes frequentemente recebem um acompanhamento rigoroso e um tratamento otimizado, o que pode não refletir integralmente a realidade da prática clínica cotidiana. Esse descompasso levanta questionamentos sobre a aplicabilidade dos achados, especialmente em subgrupos mais vulneráveis, como idosos acima de 80 anos e pacientes com disfunção renal avançada, cuja complexidade clínica pode alterar os desfechos esperados. Além disso, a abordagem retrospectiva e o período de seguimento da análise podem influenciar os resultados, exigindo cautela na interpretação das conclusões. A evolução contínua das terapias e a variabilidade dos perfis clínicos impõem a necessidade de validações adicionais em registros prospectivos contemporâneos.
