# Supplementary material for: Sobrevivência com CDI na Insuficiência Cardíaca: Realmente necessário ou Apenas um Viés de Seleção?
Source: Arq Bras Cardiol. 2025 Mar 27;122(2):e20250142. [Article in Portuguese] doi: 10.36660/abc.20250142 (PMC12040388; doi:10.36660/abc.20250142)
Supplement: Translation

# Translation: Survival with ICD in Heart Failure: Truly Necessary or Just a Selection Bias?

**DOI:** 10.36660/abc.20250142i

**Authors:** Fernanda Almeida Andrade

**Affiliations:** 1 São Paulo SP Brazil Instituto do Coração do Hospital das Clínicas da Faculdade de Medicina da Universidade de São Paulo, São Paulo, SP – Brazil; 2 São Paulo SP Brazil Escola Paulista de Medicina da Universidade Federal de São Paulo (EPM/UNIFESP), São Paulo, SP – Brazil

**Keywords:** Implantable Defibrillators, Heart Failure, Primary Prevention

In current clinical practice, the evaluation of candidates for primary therapy with an implantable cardioverter-defibrillator (ICD) requires the consideration of factors beyond left ventricular ejection fraction (LVEF) to more accurately select patients who will genuinely benefit from the device. Recent evidence suggests a decline in the incidence of sudden cardiac death (SCD) among individuals with reduced LVEF, associated with increasing heterogeneity in the underlying pathophysiological mechanisms. However, in light of advances in pharmacological approaches that effectively reduce morbidity and mortality in heart failure (HF), the decision regarding ICD indication for primary prevention has become more complex, necessitating innovative strategies for risk stratification in these cases.^1^

Technically, ICDs for primary prevention are indicated for patients with LVEF ≤ 35% and HF classified as New York Heart Association (NYHA) class II-III, despite at least three months of optimized medical therapy. However, several criticisms arise: a) the studies supporting this recommendation are over 20 years old, b) the population with reduced LVEF represents a heterogeneous group, c) different risk levels exist for ventricular tachycardia/ventricular fibrillation (VT/VF) associated with the arrhythmogenic substrate versus non-arrhythmic mortality, i.e., related to the presence of comorbidities and cardiac function decline, and d) the implementation of new treatments and improved disease management.^1 - 3^

The study by Başkurt et al.^2^presents results based on a retrospective analysis of 228 HF patients who underwent ICD implantation for primary prevention over 62 months. When analyzing mortality rates among patients with (29.4%) and without (26%) ICDs, a worse outcome (death) was observed in the control group [p < 0.05 (95% CI)], and although no statistically significant difference was found in the long term, survival was numerically better in the ICD group. This finding may be attributed to the fact that ICD implants were performed in patients with worse clinical conditions and also because these patients received regular follow-ups due to device monitoring.^2^Similar data were obtained through a meta-analysis evaluating 12 randomized clinical trials over 20 years (n = 40,195), in which the annual incidence of sudden death was 6.5% in the RALES study (the earliest study in this period) and 3.3% in the most recent study, PARADIGM-HF. These statistics indicate a 44% reduction in sudden death rates over the years [HR 0.56, 95% CI 0.33-0.93, p = 0.03].^3 , 4^Other studies have demonstrated similar findings.^4 - 7^

## Mortality predictors: The role of detailed clinical assessment

The importance of thorough medical consultations should be emphasized when evaluating these cases. Through anamnesis, potential mortality predictors can be identified, such as age, LVEF, BNP and/or NT-proBNP levels, coronary artery disease (CAD), diabetes mellitus, chronic kidney disease, baseline rhythm, HF-related hospitalizations, and NYHA >2 classification. The study by Başkurt et al.^2^identified BNP > 508.5 pg/mL (ROC: S 69% and E 69%), LVEF < 24.5% (ROC: S 54% and E 63%), age > 68.5 years (ROC: S 62% and E 62%), and hospitalization due to decompensation as independent predictors of all-cause mortality, whereas CAD was not an independent risk factor.^2 , 3^Similar findings were reported in a study by Bilchick et al.,^5^which analyzed a large cohort (n = 45,000), demonstrating that critically ill patients with multiple comorbidities have a higher risk of adverse outcomes.^5^

Therefore, the need for a risk score has been discussed and validated over time, with examples including the MADIT-II ICD Risk Stratification Score and the Seattle Heart Failure Score. However, important differences exist between them: the presence of associated cardiac resynchronization therapy (not included in the Seattle model), differences in the definition of severe arrhythmia and arrhythmic death (MADIT-ICD Benefit Score uses potentially fatal arrhythmias as a marker of arrhythmic death, whereas other studies distinguish between sudden and non-sudden death), and updated validation with contemporary cohorts. The MADIT-ICD Benefit Score, proposed in 2021, aims to improve risk stratification by assisting individualized clinical decision-making through the identification of primary ICD implantation candidates with the highest survival benefit, those whose predicted VT/VF risk surpasses the competing risk of non-arrhythmic mortality.^8^

## Limitations and future perspectives

The facts are clear: shock rates and mortality among ICD patients have decreased due to improvements in healthcare systems, HF pharmacological therapies, and easier access to physicians. The indication for ICD in primary prevention has been questioned, especially considering "new" drugs such as sodium-glucose cotransporter-2 inhibitors and angiotensin receptor-neprilysin inhibitors, which have revolutionized HF therapy. Currently, many ICD patients never receive a shock, while others experience inappropriate shocks and other complications without the anticipated survival benefit.^4 , 9 , 10^

Guidelines may require revision as new multicenter, randomized trials are conducted, particularly those evaluating populations treated with the most recent therapeutic approaches in combination. Although the recommendation for ICD implantation in non-ischemic HF primary prevention has been downgraded in the latest guidelines, it remains a Class I indication for patients with ischemic HF. In studies supporting ICD benefits for primary prevention in this population, patient stratification was performed using electrophysiological testing, and adherence to optimized medical therapy (defined in guidelines as the use of beta-blockers, ACE inhibitors/ARBs, and aldosterone antagonists) was limited; even today, international guidelines do not mandate the use of "new" drugs as a prerequisite. However, routine use of pre-implant electrophysiological testing is not part of standard clinical practice. The MADIT-ICD Benefit Score emerges as a tool for shared decision-making between physicians and patients, providing a personalized and integrated assessment of the competing risks of VT/VF versus non-arrhythmic mortality in candidates for ICD therapy.^1 , 9 , 10^

In conclusion, data from multicenter randomized clinical trials represent a significant advancement in understanding ICD benefits. However, it is important to acknowledge that participants in these studies often receive rigorous follow-up and optimized treatment, which may not fully reflect real-world clinical practice. This discrepancy raises questions about the applicability of findings, particularly in vulnerable subgroups such as patients over 80 years old and those with advanced renal dysfunction, whose clinical complexity may alter expected outcomes. Additionally, the retrospective nature and follow-up duration of some analyses may influence results, requiring caution in interpreting conclusions. The continuous evolution of therapies and variability in clinical profiles necessitate further validation in contemporary prospective registries.
